# Causal evidence for social group sizes from Wikipedia editing data

**DOI:** 10.1098/rsos.240514

**Published:** 2024-10-02

**Authors:** M. Burgess, R. I. M. Dunbar

**Affiliations:** ^1^ ChiTek-i AS, Oslo, Norway; ^2^ Department of Experimental Psychology, University of Oxford, Radcliffe Quarter, Oxford OX2 6GG, UK

**Keywords:** trust, social groups, group dynamics, wikipedia, collaboration

## Abstract

Human communities have self-organizing properties in which specific Dunbar Numbers may be invoked to explain group attachments. By analysing Wikipedia editing histories across a wide range of subject pages, we show that there is an emergent coherence in the size of transient groups formed to edit the content of subject texts, with two peaks averaging at around N=8 for the size corresponding to maximal contention, and at around N=4 as a regular team. These values are consistent with the observed sizes of conversational groups, as well as the hierarchical structuring of Dunbar graphs. We use a model of bipartite trust to derive a scaling law that fits the data and may apply to all group size distributions when these are based on attraction to a seeded group process. In addition to providing further evidence that even spontaneous communities of strangers are self-organizing, the results have important implications for the governance of the Wikipedia commons and for the security of all online social platforms and associations.

## 1. Introduction

Human social groups have a hierarchical structure whose layers have very specific sizes [1]. They form a harmonic series at which the information flow around a social network is optimized [2,3]. The layers emerge owing to both the frequency with which individuals contact each other [4,5] and the sense of trust they have through increasing familiarity [6]. The evaluation of cues for community membership, known as the Seven Pillars of Friendship [7], and the exchange of tokens have previously postulated this [8,9]. The base of the group hierarchy lies at approximately five individuals. The upper limit at which individuals have been found to take part in a coherent conversation [10–14] is 4, which appears to be determined by the cost and capacity for humans to manage the mental states or viewpoints of other individuals [12].

Experiments to scrutinize these notions are notoriously difficult to perform at scale; however, the community structures surrounding Wikipedia have provided us with a serendipitous opportunity for a direct test of these claims, since they gather and publish data in a completely open manner. In the course of studying Wikipedia in relation to matters of online trust, we stumbled across a signal in the data concerning the dynamics of groups that we report on here [15].

Individuals come to the Wikipedia platform with a variety of motivations that ultimately result in the editing of Wiki pages. This is a very well-documented online process performed by large numbers of individuals, who may or may not be aligned in their intent. It is one of the few online platforms or ‘virtual societies’ that openly shares the histories of editing interactions on its pages. There are several classes of agents involved in editing. About 20% of changes are made by editorial ‘bots’: these perform administrative functions like labelling, fixing citations and correcting spelling, etc. Such change bots have different tolerances and limits to humans. Editors are usually strangers and often appear to other users as confrontational figures. This allows us to test whether the self-organizing principles that structure personal social networks also apply to groups of strangers and, by implication, to groups formed for the sole purpose of work. Our study seems to show that the emergence of highly predictable group sizes depends entirely on the existence of an initial seed event as an attention attractor, and an accounting of the economics of that attention as part of the phenomenon of trust. Evidence of conflicts at the user level have been considered before [16]; however, we are able to approach the issue of contention with greater universality to eliminate specific biases.

Wikipedia has a wealth of pages spanning a large number of subjects. These can be viewed as evidence of a wide variety of intent among users. Averaging results over large numbers of topics would therefore be expected to erase any biases associated with specific subject material, leaving only properties that characterize common aspects of human participants. Such characteristics would likely be innate to humans, since the collection of contributors is broad and internationally diverse thanks to the Internet. In other words, Wikipedia samples contributions from a wide variety of individuals with different motivations leading to a high level of independent variability (entropy) in the writing pool. This grants us the study of the universal aspects of group phenomena with statistical credibility.

The study we report here is thus part of a broader study on the topic of trust. It reveals an emergent coherence in the sizes of transient groups, which coalesce to edit the content of the Wikipedia pages. The results demonstrate good agreement with earlier predictions about social groups. Groups on Wikipedia come together episodically and average at around N=8 persons during an episode. The probability for a new user to join a group peaks at around N=4 and the distribution has a long exponential tail. We have obtained a simple formula for this probability distribution of transient group sizes [9].

## 2. Data collection and analysis

We analyse the statistical mechanics of the time series of changes, recorded by the Wiki platform, as an underlying causal process and use very general arguments from dimensional analysis to gauge the dynamical behaviour of users in the limit of a large ambient population [17]. In the interests of focus, we shall sketch only the most pertinent details from the full experiment, i.e. those which contribute to the main result of this paper summarized by the formula in equation (4.2) and the data fit shown in figure 5 below. Full and detailed descriptions of methodology, process, data, as well as computer code are shared freely online by Burgess & Dunbar [18], and further discussion about the theory may be found in Burgess [19].

The principal source of data for user groups comes from studying the change history logs, which are maintained automatically and impartially by the platform software, one for each Wiki subject page. Analysis of these text logs parsing their ordered timelines is quite involved and somewhat time consuming. It involves parsing and extracting data iteratively. The ultimate outcome is a multivariate time series for each subject page.

In order to cover a broad mixture of users and subjects in the data, a selection of Wiki page subjects was chosen randomly to form a base list of subjects; this was then sub-sampled in batches (in the manner of a Monte Carlo method). Answers from partial data were assessed for stability under varying selections; this helped to keep repeated experiment executions to within reasonable time limits and establish repeatability.

Initial tests to determine a suitable ensemble size with statistical stability resulted in a sample set of around 800 subject pages, which was then sub-sampled in groups of 100. These pages, in turn, involved interactions with around 200,000 separate online contributor identities. The results, while still noisy by a few per cent, exhibit a robust statistical stability (as shown by the error bars in figure 5 below). Our final results are based on the full merged set of data for completeness. A wide variety of variables could in principle be extracted from Wikipedia data. We focus on just a few to demonstrate the main result of the study. Table 1 shows the quantities used to assess the dynamics of the user behaviours.

**Table 1.** Key quantities in the time series analysis.

L	the current total length of the Wiki article being edited in characters
N	the number of users associated with an ‘episode’e
$\Delta \,t_{i}$	the UTC clock time interval at which an edit was reported
$\Delta \,t_{i}=t_{i}-t_{i-1}$	the elapsed time interval in seconds between edit i andi−1
$t_{i}\,\left(e_{n}\right)$	a time stamp identified as belonging to an edit from episode $e_{n}$
I⁢(N)	the number of contention markers counted during the episode

Each article editing history amounts to a time series of data, time stamped with a UTC clock value and annotated with editing measures and human comments. The arrival times for edits are ‘bursty’ and fall into clusters that we identify by looking at anomalous arrival times. An episode is a cluster of edits separated by a notable cessation of editing (see below). We look for variables that offer evidence of users interacting with one another punctuated by periods of rest. From the theory of trust, we expected contentious interactions between users to be the significant driver of activity. Previous work has looked at conflicts in Wikipedia editing with results that align well with ours, albeit with a different focus [16]. Contention is indeed evident qualitatively in the data. In particular, users do not appear to be attracted to working together cooperatively, but rather tend to come into dispute with one another. Such contention between user identities can be identified by evidence of actions such as tit-for-tat editing, trigger words in the comments and so on. The contention intensity function may thus be defined as a piecewise integral of discrete signals from the history text and difference data:


(2.1) $$\begin{array}{r} I_{e}(t_{i})=\left\{ \begin{array}{c} \text{undos of previous edit}\mapsto +1\\ \text{signal words in comment}\mapsto +1 \end{array}\right. \end{array}.$$

Signal words refer to phrases in editing logs that typically identify complaint about an entry, include ‘citation needed’ and ‘vandalism’. Some of these remarks are added by bots for consistency. Undos could be established as explicit reversals and others by the size of an edit on location. While these inferences are approximate, they are unambiguous signals of increasing contentiousness, giving a score of one or very rarely two for an edit. The count is summed over times relating to an episode, starting from $t_{i}$ and finishing at $t_{f}$:


(2.2) $$\begin{array}{r} I=\int_{t_{i}(e)}^{t_{f}(e)}\;I_{e}(t)\;dt \end{array}.$$

**Figure 1.**
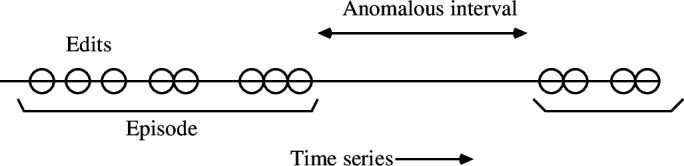
Episodic bursts are defined by identifying an anomalous cessation of editing in the running average of interarrival times between edits. A new episode cannot be less than a day apart but must be more than an order of magnitude greater than the running average.

The episodic or bursty nature of editing implies that users have an inconstant work rate, rather than making steady plodding increments. A key issue is therefore how to measure a timeline of such variable rate interactions (see figure 1). Absolute work output measures time (as one sees by examining the length of text produced L). Also, time stamps encoded in change logs measure an embedding time. Since the discussions are asynchronous dialogues (i.e. not face to face), one cannot infer too much from the precise clock intervals between responses. A response to one edit could take weeks or minutes. Rather we look for indications of continuity or discontinuity of process. Every process thus has its own clock; to assess a process ‘proper time’, we used the intra-edit interval $\Delta t_{i}$ and its running average to look for significant pauses, defined as an absence of editing of an order of magnitude greater than the running average, and this was then used to identify a scale for episodic cohesion. A local running average was used to adapt to the inhomogeneous rate of editing and also because there is no natural normalization scale for a global average between documents.

A local punctuation scale was introduced to dissect and splice the timeline into episodes, in equation (2.4). If the intra-edit interval was suddenly much greater than a normal response time, $\Delta t_{i}\gg \langle \langle \Delta \rangle \rangle$, i.e. by an order of magnitude, then we consider that one episode was ended and another was begun: $e_{n}\mapsto e_{n+1}$. Episodic time was thus identified by the separation of scales measured in UTC time: where,


(2.3) $$\begin{array}{r} t_{i}(e_{n})\gg t_{f}(e_{n-1}), \end{array}$$

i.e. by a punctuation interval which is large compared to the interval between successive interactions:


(2.4) $$\begin{array}{r} \Delta t(e_{n},e_{n-1})=t_{i}(e_{n})-t_{f}(e_{n-1})>\Lambda \;\langle \langle \Delta t_{i}\rangle \rangle . \end{array}$$

This is what one would expect in the physics of work: the work rate or ‘energy’ of attention (which is associated with kinetic trust, or the investment of attention given to process sampling, as defined by Burgess [6]) should be associated with the timescale of attention processes. A dimensionless order of magnitude scale factor Λ∼10, used as a multiplier of the expected interval between edits (denoted $\left\langle \langle \Delta t_{i}\rangle \right\rangle$), defines for us a notable cessation of activity. In addition, a minimum $\Delta t(e_{n},e_{n-1})$ of 1 day between episodes is enforced to remove artificial bursts faster than a normal human attention span. To adapt to fluctuating editing rates (user edits are not undertaken on a regular clock basis), a simple convex running average was defined by the recurrence relation:


(2.5) $$\begin{array}{r} \langle \langle \ldots \rangle \rangle \;:\;\langle \langle \Delta t\rangle \rangle_{i}=\rho \;\langle \langle \Delta t\rangle \rangle_{i-1}+(1-\rho )\Delta t_{i}. \end{array}$$

One is essentially looking to define a ‘fair weighted’ adaptive scale for averaging, given inhomogeneous time series of inconstant length. This is a very simple approach. A memory retention rate ρ=0.4 was used to favour rate changes within a few interactions [20].

Edit episodes were obviously not of equal length, especially on the strict wall clock time, but each could be measured in the characteristic timescale of the page process itself. The latter was measured by searching each process for bursts of change and intervals of cessation, leading to a characteristic timescale. The correct identification of process time is necessary to scale activity dimensionally in accordance with dimensional analysis and the Buckingham Pi theorem [17]. This method is a standard way of eliminating variability in the magnitude of statistical fluctuations. For the finite datasets, the episode’s clock rates between activity and inactivity fall into separable bands with an easily computed average value. The parameter β in equation (4.2) serves as a residual ‘manual knob’ with which to adjust the efficiency of this coupling.

Apart from the identification of episodes as a temporal phenomenon, our other key variable is the size of groups associated with these meta-events. For the avoidance of doubt, the number of users in a time series can be defined with turgid precision by an idempotent sum over distinct user identities over an episode:


(2.6) $$\begin{array}{r} N=\text{dim}\;\overset{t_{f}(e)}{\underset{t_{i}(e)}{⋃}}\;\text{username}(t_{i}) \end{array}.$$

It is unknown how many different identities real persons might have, but this will not affect the analysis as long as no user impersonates more than one individual during a particular editing episode. The number of edits per episode is not a relevant measure here, but is represented indirectly through the contention numbers. Furthermore, there is no measurable role played by the subjects themselves in biasing the results: a page about controversial politics was not observably more contentious on average than a page about sober mathematics. This idea was tested across random samples of all topics. The only constant in Wikipedia editing was a tendency for contention to arise, regardless of the subject. By eliminating the times from these quantities, we can represent the contention as a function of the group size I(N) per episode, with other factors ignored, and further consider the distributions of these.

Once it became clear that edits involved finite episodic groups of users, we need to know whether groups were formed by interactions of order $N^{2}$ or of order N unique users. If group members had no preferred member, then one would expect a closer fit to a complete clique of order $N^{2}$. Conversely, with an interaction seeded or led by an initial individual (a strong leader or just someone who starts the ball rolling), the process of group attraction would be a star network of order N. Order N variance was markedly less than for plotting against $N^{2}$, suggesting that once an episode has begun, the episode itself acts as an attractor for uncoordinated individuals to arrive and join in. This was even more strongly evident in the shape of the probability distribution. A dependence on N rather than $N^{2}$ is reflected directly in equation (4.2), based both on theory and empirical fit.

**Figure 2.**
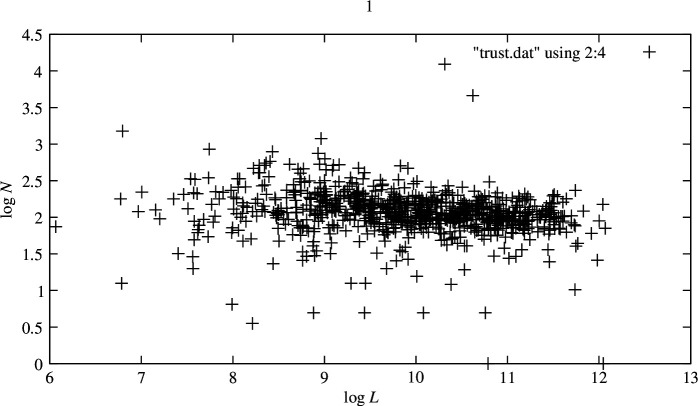
Relationship between the average number of users contributing per episode as a function of article length. ⟨N⟩(L) is also noisily constant or decays in a weakly exponential manner, suggesting that there is a more or less constant stream of new battles to be fought or issues to be solved. The (natural) log plots show a weak tendency to exponential behaviour, as expected from a probabilistic arrival process. However, the restricted number for N evident in all the graphs makes this almost insignificant.

There are several checks we can undertake to verify the consistency of these choices, including measuring the correlation between text length L and the total number of users counted in the log for each article to see if some users are responsible for most of the text or not. Although this does not necessarily affect the history of interactions surrounding the editing, it speaks to the potential biasing of results around specific ‘strong’ individuals. The log–log plots in figure 1 show this relationship to be a noisy power law, taken across all the articles. A fairly constant rate of users is involved per article length. The average duration of an episode expended per number of involved users is plotted in logarithms in figure 2. There is thus a rough correspondence between the length of a Wiki article and the total number of users involved in its creation, as one might expect both to be measures of process work (effort). Conversely, the level of contention between users was quite independent of the subject matter. Our measure of contention in the editing process is flagged both by argumentative trigger words in the log text, as well as direct actions such as undoing a previous user’s efforts combined into a single counter.

We point out that the subject matter of Wiki pages was at no time relevant to the group dynamics. We were able to infer that—over the total sample—subject matter acts mainly as a seed for attracting certain users. Once these users arrived, they tended to behave quite uniformly. If one delved into the psychology of individual responses one might find hidden depths to interactions, but on a statistical level behaviour is ruled by simple signalling of an intent to engage contentiously. We saw no particular correlation between user behaviour and subject matter. There is as much contention for subject pages on cabbages or mathematics as for those on politics and celebrity adulation, provided they were sufficiently long lived. This is presumably because the reasons for contention and criticism are human-led qualities and thus somewhat similar across all subject areas. More interesting, though not surprising, we found that human behaviour differs from machine bot behaviour in this regard. Excluding machine contributions (which are easily identifiable by name) brought the probability distribution closer to the predictions made in earlier work. Including them led to a small shift of supportable group sizes, as machines can tolerate contention better than humans. In summary:

— We find that a high level of variability in page characteristics and contributors is consistent with sampling invariance and use this to adopt a Monte Carlo approach, sampling random groups of pages and averaging the results. Since analysing the pages is time consuming, even for a computer, this allows us to infer a high confidence in the consistency of results from independent random samples. We then derive the final results from 827 subject pages and just over 260,000 involved users.
— Users come to edit a subject page that they care about more or less ad hoc. Once one author has arrived, others will notice the changes and join in. These begin to form a group to either challenge or improve on one another’s contributions, depending on the alignment of their intentions.
— Editing takes place in bursts of activity, punctuated by longer gaps. We can thus identify a series of ‘episodes’ for every page, which we take to be causally independent. Each episode yields a group size and duration and a level of contention that one sees from the signals embedded in the historical record.
— For every subject page, there is also a history page which we can read and analyse using appropriate software. The pages show evidence of user identities, which might be transient and even deliberately anonymous but which are assigned regular identifiers by the platform. We can see which edits were contested by others and the sizes, dates and times of the edits. This allows us to construct a process timeline and to identify contention between individuals. Users can argue and even undo one another’s changes, and these events can be counted.
— There is little evidence of coordination between the contributors across episodes. Coordination is essentially a stigmergic process through the medium of the editing produced. In part, it is also a confrontational process, as we see from the history and discussion pages.
— Contention between users can be identified when one user undoes the contributions of another, rather than adding to them. From even a cursory inspection of results, it is clear that contention between users is a significant issue in editing on all pages, so special focus was attached to the level of overlap between edits and on cases where one user undid the changes made by another.
— We also note that, in excess of 20% of all edits were made by automated ‘bots’, some of which are designed to patrol changes for such activity (as well as trivial issues like spelling corrections), so the intentional aspects of behaviour are to some degree invoked by proxy.

## 3. Dynamical factors in Wikipedia editorial clusters

Figure 2 shows how the number of users involved per editing episode varies with the length of the main article at the time of snapshot. The article length may be taken as a heuristic proxy for the total time invested by all editors of the page’s history. We see that the rate of user comings and goings decays almost imperceptibly with the elapsed time or length, suggesting a slow stabilization of subject pages. However, this is small compared to the level of activity maintained over time. In essence, we see little evidence of pages ever being finished.

All the pages we examined appear to be subject to continuous contentious editing. The rate of stabilization with length (time) is quite slow, as can be seen using the text length of the article as a proxy for total time elapsed since creation. The bursty nature evident in the data suggests that causal behaviour is limited to individual episodes, so we treat each episodic burst as a separate event.

**Figure 3.**
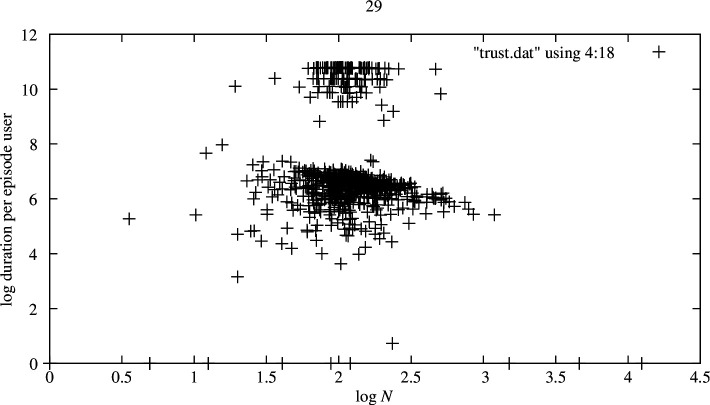
Duration (lifetime) per episode versus N in rescaled episode time. This is the average fractional time spent by a single user over an episode. The duration is measured in days. The graph axes (natural logs) all show clustering in a region, rather than a line relationship, suggesting that the tendency to continue interacting (fighting or discussing) decays weakly and exponentially with the number of users, but is almost constant. The number of users itself does not vary much in episodes, so the cluster is narrow.

The lifetime over which a group edits is closely bounded in duration, although the bounds are very noisy. Figure 3 shows the existence of at least two distinct classes of episode duration evident in widely separated clusters, but these are not distinguishable by our counting method. This is not an artefact of the analysis: the same feature recurs in different sample sets, although most of the activity is in the lower band. In effect, the size of an active editorial group remains surprisingly constant despite considerable turnover in membership over time. It remains to be studied whether there is significant turnover in the groups with very long durations. This would tend to make sense, given that the size of the group remains statistically constant over even exponentially longer interactions.

From the N dependence of data, we infer that users are attracted to an editing episode by the initiation of changes made by a user seed. In other words, some user comes at random to perform work and that attracts others to see what’s going on. The number of users in a typical episodic event is noticeably clustered. We see this in a few measures, the most interesting of which is the plot of contentious changes versus size of users in an episodic cluster. An average group size associated with contention is around N=8 (with a variation between 7.75 and 8.2, depending on how we calculate and sample the average). We take this to be the group size at which contention reaches its maximum limit. Note, however, that upwards of 20% of all users on the platform were approved bots working by proxy on behalf of both the platform and others. If we exclude bot activity, there is a small reduction in average group size (from 8.2 to 7.6) that brings the data fit closer to the numbers widely discussed in the literature.

**Figure 4.**
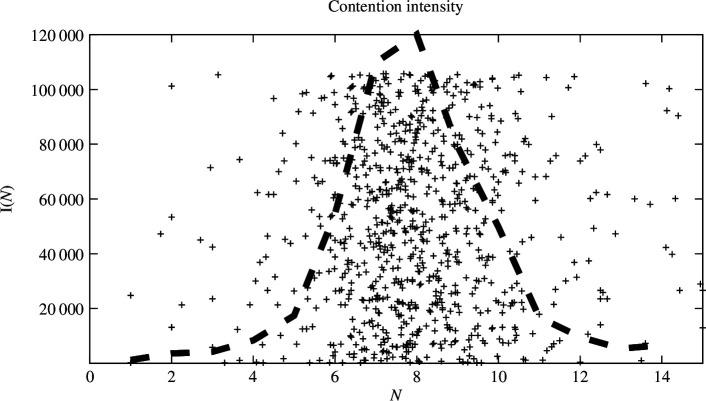
The contention I(N) or intensity of mistrust, peaks around N=8 (with a variation between 7.75 and 8.2). The scaled probability density for the raw data is superimposed as a dashed line for visual aid. A more nuanced value for N can be found as an expectation value of this distribution to account for the uncertainty. The vertical cutoff in history length accounts for the flat top in the data point ceiling.

Figure 4 shows an enlargement of the non-empty region of the plot of contentions per event as a function of users per episode. This shows that, although the rate of contention varies somewhat between subject pages, the size of the group and the level of contention is confined to a relatively predictable region of parameter space in the range N=[0,15], centred on N=8. There are no other clusters evident. This tells us both that contention is intrinsic to users and that users form groups of predictable size. This suggests that there is a single causal explanation for this cluster size.

**Figure 5.**
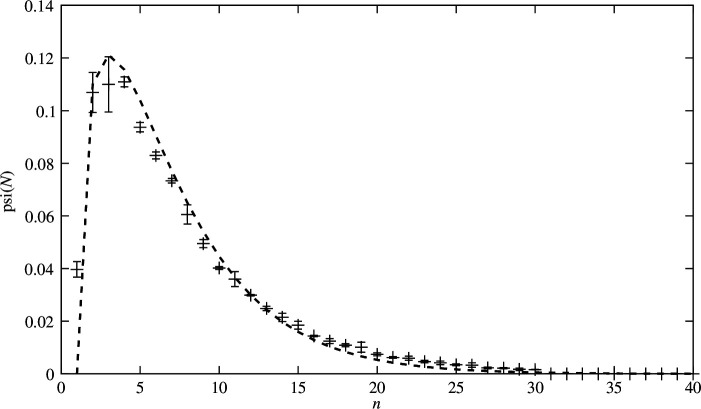
The principal result: a normalized frequency spectrum of group sizes indicates the probability of finding an episodic group of size N. We note that the peak near N=4 is well below the group size that maximizes contention at N=8.8. The dashed line shows a theoretical fit for the curve derived from theory and dimensional considerations (see text). Crosses indicate estimated error uncertainty. Even small changes to the functional form of this curve lead to significant deviations from the data, which is suggestive that the theory is basically sound.

The most striking evidence of group size coherence may be seen in the frequency plot, however. In figure 5, crosses show actual data and the dotted line a theoretical fit. The unexpectedly close fit suggests an underlying determinism, albeit on a statistical level. The spectrum of group sizes across all episodes is remarkably free of the noisy artefacts from specific measurements, so we expect it to be quite robust. Error bars are no larger than the crosses. It has a classic decaying exponential tail typical of the logarithmic scaling of maximum entropy processes. More significantly, it also has a non-exponential growth feature for small N, which is where we expect the interesting dynamics to be.

Taking different sample sets at different times and of different sizes had a negligible effect on the values for the averages used in the scaling relation, as one would expect for universality relations. Clearly, the noise levels could vary. However, since we rescale by average noise scale in order to compare unequal processes, this is largely eliminated. Such rescaling is typical of how one eliminates noise in statistical mechanical formulae out of equilibrium.

The implications of this for group sizes is that universal characteristics do not depend on the details of each process but rather on the core rates of interaction specific to the agents involved. Only by eliminating time as a variable can we hope to compare the innate properties of widely different agents according to their core commonalities. We speculate that those would be the cognitive or interaction process invariants, and the order of magnitudes of the ratios seem to confirm that both represent typical sociological and neurological values.

Note that our analysis was not trying to optimize a regression fit of pre-classified data onto an off-the-shelf distribution, as one might hope to do for a known process like a questionnaire. Human conversations, after all, are not processes based on completely random variables. Given what we could not know, we have kept to somewhat conservative methods, principally using dimensional analysis to look for scale-independent effects, which might be compared to theory [19]. There is no other reason to expect a cleanly functional form for the process of episodic interaction.

## 4. A model of group formation based on mistrust

We were able to explain the results in terms of a model about the dynamics of agents underlying trust. This combines the roles of user intent and the work effort involved in coming together. Readers can find a comprehensive discussion of the physics of this model by Burgess [6,15].

To do this, we measure the amount of semantic ‘novelty’ or ‘attention provoking content’ in both the Wikipedia article texts and the supporting editing histories. We define the amount of work done by users according to the length of their text changes in alphabetic characters (for glyphs we can also count the numbers of strokes), since short or easily written words are generally more common.

Since we know that users join episodic groups coincidentally, randomly searching and only later following if they are sufficiently attracted to participate (they do not receive notifications or marching orders), the story suggested by this spectrum is that users accrete into groups ‘gradually’, based on subject and activity. On reaching a certain critical size they then fizzle out. The timescale for this process is not constant, but we are rescaling the episodes according to the average rates for each subject, as measured in advance.

We see that, initially, the probability of adding new users to a group begins to falter around a maximum of N=4. In other words, users are less inclined to join a discussion that already contains four persons. By the stage at which contention is maximal (N=8), the probability of new attachments is already significantly reduced. Since we know (from the timeline data) that the group episodes terminate, we conclude that the entire group disbands. Thus, the groups themselves decay over their proper time durations, probably as a result of general diffusion of averages due to mixing. Such exponential decay is a typical statistical feature of broad mixing (for further details, see Burgess [19]).

The amount of work expended by users is linked to the amount of attention offered by each agent to the conversational interaction, not to the amount of text added. This fact plays a key role in explaining the results in terms of mentalizing and mistrust. The amount of activity invested appears stimulated by what we might call mistrust of the changes made by others rather than the amount of it. We can assume that few if any of the agents know one another. There is certainly little evidence of explicitly cooperative behaviour. Over time, one expects all text might be altered in major and minor ways, but when this occurs in the manner of a duel between two users, it marks a significant semantic interaction. In such a case, we characterize the behaviour as ‘contentious’.

Let us assume that there is a reservoir of agents whose intentions are distributed with high statistical variation (entropy). Wikipedia does not advertise or incentivize editing in any way, so the arrival of a single agent to edit a page is completely ad hoc. We can assume that a topic is represented by a direction in the space of subjects and that there will be a subset of the agents who are aligned with their understanding of this approximate topic. The arrival and departure of users now indicate a kind of ‘detailed-balance’ model for the statistical stability of the group spectrum, i.e. the probability of finding a group of size N. We can assume, from the earlier remarks, that the set of users affiliated with the Wikipedia platform is large and broad in its characteristics, i.e. it has effectively maximum entropy at large N.

Suppose that a new Wikipedia topic page is started at random by any user or agent. Eventually, another agent will find the changes and be attracted by the seed of a common interest. If users trusted one another there would be no need to look, but if they don’t then they invest time to verify and possibly alter the text. This hub attraction leads to kinetic work activity proportional to N−1. To determine the rate of attention for the agents, the trust model by Burgess [6] follows the guidance offered by the dimensions of work/energy for work done by a pressure or force and the resultant kinetic response. Precise details needn’t be known to find the rate of temporal evolution as a velocity change from the kinetic work ($1/2mv^{2}$). A schematic model based entirely on work exchange has been given by analogy with Boltzmann processes by Dunbar [21], with similar results to ours; however, we can predict the precise parameter values to give the fit in figure 5.

Given any level of attractive potential for curious agents, defined as a trustworthiness assessment [6], the rate of kinetic attachment would be expected to flatten like the square root of that potential investment of work. From information theory, maximal entropy in a conservative reservoir of agents generates a Boltzmann distribution exp⁡(−βE), where E is a work-energy parameter. We don’t actually require the number of possible agents to be conserved, as the number is assumed to be essentially infinite. A parameter β represents the agents’ average intolerance for contention ‘back pressure’. Choosing the only dimensionless combination of parameters ν∼β(N−1)/⟨N⟩T, where N−1 is the group size exerting pressure on a newcomer and ⟨N⟩T is contention maximum groups scale, we obtain a probability of


(4.1) $$\begin{array}{r} Pr(N)=Pr(\text{attachment})\;AND\;Pr(\text{dissipation})∼\sqrt{\nu }\times \exp(-\beta \nu ). \end{array}$$

From this, we can derive an expression for the probability of finding a group of size N:


(4.2) $$\begin{array}{r} \psi (\nu )=\frac{4}{\sqrt{\pi }}\;\frac{\nu^{\frac{1}{2}}\;e^{-\nu }}{\langle N\rangle_{\bar{T}}},\;\nu =\frac{2\beta (N-1)}{\langle N\rangle_{\bar{T}}},\;(N>1). \end{array}$$

The broad narrative implied by this result is that new pages are random events (‘event one’ of an episode). These events form a seed that attracts the attention of others. In a group of total size N, N−1 will be stimulated by the seeding to follow the rising square root rate of kinetic attention. This corresponds to ‘grooming’ work in the language by Dávid-Barrett & Dunbar [22]. As the back pressure arising from inevitable contention in the group rises, innate limitations on agents’ capacity for this work drives them away. The variability of the total mixture ensures this is exponential on a large scale.

The square root growth term is a consequence of the work/energy invested in attending to the ongoing process. The cognitive cost of increased familiarity is an increased processing time cost [23]. In physics, energy is the complementary variable (and thus generator) of temporal evolution in a system. This relationship is essentially information theoretic on a statistical level. In this respect, information is a cybernetic concept in the sense that it can involve the exchange of actual information about some aspect of the world or some token such as grooming that defines the quality of a relationship (see also West *et al*. [2,3]). The link between intentions, promises and trust points to this intentionality as the seed attractor in the explanation of process network dynamics [15]. The attractive ‘force’ during growth is not a network effect but a kinetic attention effect over these networked bonds. Attraction does not therefore necessarily imply proximity in physical spacetime, but rather in intention space (i.e. the alignment is in intention first and in position only as a secondary consequence of attention [15]).

We need to be careful in interpreting statistical distribution laws as causative, because a statistical law does not necessarily have reverse causal implications for individual instances. Indeed, we can note that equation (4.2) has the form of a Gamma function, such as that found by Dunbar [21] from generic arguments of statistical mechanics. We believe that it is the universality (in a statistical sense) of the phenomena that allow the models to agree. Ours is based on causality and direct investment of aligned intent, theirs is based on analogy to work done by aligned momenta in an ideal gas leading to a Maxwell-Boltzmann distribution [21]. This has a passing dynamical similarity to our case of humans interacting, but no direct connection. Wikipedia edits are by no stretch of the imagination an ideal gas. In our case, a tolerance for group sizes derived statistically may only be a weak indicator of individual behaviour; however, there is something intriguing about our specific result on a number of levels. There is an ‘invisible hand’ style shaping of outcomes which is not quite a force, but which could be modelled as one in an effective theory, like the alignments of momenta corresponding to the alignments of intentions. Indeed, theory suggests that there is a relationship between these outcomes, intentionality and the innate capacities of the agents [6].

The distribution in figure 5 relates the tendency for contention in a group to a tendency for neighbours to tolerate one another’s presence. Initially, this work seems to be the very essence of the process of discussing and contending over subject matter editing changes. Later, this progress seems to be overpowered by the tendency for the costs of contention to overcome the perceived benefit. This interpretation makes sense because the contention maximum always lies at larger size than the most common maximum size. In other words, contention is still growing when the group starts to falter.

## 5. Universality of the distribution and bots

It is tantalizing to speculate on the scaling implications and the wider behaviour of the curve in figure 5. Implicit in its functional form is a kind of stepping stone hierarchy of scales, determined in detail by the contention cost β. Although we have only obtained data here for ⟨N⟩T=8, we can nonetheless extrapolate the consequences of the model for other values with data from other sources. If we examine the relationship between the maximum of contention and the maximum equilibrium group size, we see an interesting hierarchy of sizes, reminiscent of the Dunbar hierarchy (5, 15, … 150, 500, etc.). The precise values depend on the choice of β, whose value lies close to 1 for this work, but tolerates minor adjustment to smaller values. The value that generates the usual Dunbar hierarchy of sizes lies between 0.875 (without bots) and 0.95 (with bots).

Implicit in the statistical law defined by equation (4.2) is a prediction that there is a fission rate for groups with contentious interactions of approximately 2β for N≫2β (for smaller N the integral nature of N precludes a precise analytical expression). The values in this study are consistent with the work on conversations summarized by Dunbar [24]. These show conversational groups of around N=8 rapidly partitioning into two separate semi-independent subgroups. The emergence of a contention limit or peak level plays the role of an ‘innate’ characteristic for the bulk of the agents. We should not forget that 20% or more of agents are in fact software bots, with potentially infinite tolerance, whose behaviour is largely triggered by human activity. The role of attractors is a key feature, represented by the factors (N−1) in the spectrum.

In the case of the β=0.875, we have a series close to 5, 15, … 150, etc. A group that could loosely contend at n=550 would tend to break up into fragments of 150, and a group of around 15 might break up into smaller groups of order 5 under pressure and so forth. This suggests an interesting kinetic link between the dominant group scales. Again, we emphasize that these are statistical tendencies not causal rules at the network level. The network causality implicit in the formula is one based on the kinetic work done by the process supported by the local network. The precise numbers are inevitably subject to a degree of statistical uncertainty, but the essence of the story is compatible with the scaling hierarchy of Dunbar numbers known from wider sources [1]. Removing all bot interactions from the data alters average N slightly to give an effective value of β=0.93, which is closer to this idealized series.

## 6. Discussion

The signature of an apparently universal group dynamics is evident within the data mined from Wikipedia. Discovering such a beautiful statistical curve, as shown in equation (4.2) in a human process, is a rare opportunity. The difficulties of meaningful reproducibility faced by the social sciences make it difficult to find innate phenomena that can rival those of physics or chemistry. While some authors have argued by analogy to obtain parameterized fits, we are able to offer a causal model that has no free parameters but provides excellent agreement with data. We find a remarkably robust expression of scaling. We did not set out to look for it, yet it seems to dominate the behaviour of contributors and offers a fortuitous opportunity to measure details normally not available to onlookers.

We should be clear that, while our theory might have wider implications, the data we analyse here only provide evidence for a single phenomenon, with characteristic scales $n_{\text{max}}≃4$ and ⟨N⟩T≃8. Nonetheless, the scaling treatment suggests a universality that might extend to other groups, too. Natural conversations have an upper limit of four members [10–14,21], with this limit seemingly set by the mentalizing capacities of the average adult [12]. Other processes that follow the same *dynamical similitude* could be viewed as analogous. We discuss this elsewhere [19].

The question we are left with is what is the seeding force or ‘invisible hand’ shaping attraction and repulsion in the group processes. Is it the charismatic leader, the threat of predation (animal or human), the need for cooperation, etc? The formula suggests there will be one, eventually represented by the proxy of the group itself. For Wikipedia, it is clearly the suspicion that someone is changing a subject others care enough about to defend. In this sense, the process of ‘grooming’ or maintaining the relationship is with the subject matter rather than the others in the group directly, and it is clearly a kinetic one: the activity is that of a standalone agent, which is therefore limited by the characteristics of the agent, some of which are innate and others a function of state.

One of the motivations for studying user behaviour in informatics is to understand the security of online platforms such as Wikipedia. Such platforms are increasingly prevalent features of our lives. In Informatics, trust is usually handwaved away with very simple questions about identity reliability (trustworthiness), leading to misleading calls for ‘zero trust’ behaviour. Trustworthiness is a trait that an agent or individual may have in some quantity (usually varying probabilistically between 0 and 1) that characterizes you as an individual [24]. Trustworthiness (usually recognized by cues of various kinds) can act as a ‘first pass’ basis for action, but it is at best a broad-brush cue based on generalities. Trust, on the other hand, is not a trait but a process that is built up over time between two individuals through repeated interaction: I trust you because I have interacted with you on a number of occasions and found you to be reliable (as an individual). The challenge in using trust as a characteristic in the security of systems is that it represents something taking shape over very large numbers of events. This may well make it useless as a practical tool unless there are truly universal cues that transfer from one case to another.

There has been a tendency to look to ‘complexity science’ to explain phenomena that relate to biological and social systems. We see little evidence of that in the results here. At the scale of bulk measurement, we find remarkably linear stability. This is an indication of a separation of timescales between interior processes involved in relationship maintenance and the exterior ‘boundary conditions’ of the social network. What is challenging for monitoring systems in general is that the timescales over which significant changes may occur must be faster than the timescale over which statistical learning takes place. This is surely why attentiveness is so expensive and group sizes are constrained in a hierarchy of tradeoffs. Indeed, if we seek to compare singular interactions to an average profile of bulk behaviour, we are faced with a learning challenge if we are to weed out bad actors. This challenge is precisely the reason why brains evolved their phenomenal capabilities in the context of ever larger social groups. Not surprisingly, it is currently under scrutiny in connection with machine learning for artificial intelligence [25].

## Data Availability

The data and code files are available in the specified DRYAD repository [26].
